# Quantifying the Intrinsic Strength of C–H⋯O Intermolecular Interactions

**DOI:** 10.3390/molecules28114478

**Published:** 2023-05-31

**Authors:** Jiří Czernek, Jiří Brus, Vladimíra Czerneková, Libor Kobera

**Affiliations:** 1 Institute of Macromolecular Chemistry, Czech Academy of Sciences, Heyrovsky Square 2, 162 00 Prague, Czech Republic; brus@imc.cas.cz (J.B.); kobera@imc.cas.cz (L.K.); 2 Institute of Physics, Czech Academy of Science, Na Slovance 2, 182 21 Prague, Czech Republic; czernekova@fzu.cz

**Keywords:** hydrogen bonding, noncovalent interactions, CCSD(T), DFT, SAPT

## Abstract

It has been recognized that the C–H⋯O structural motif can be present in destabilizing as well as highly stabilizing intermolecular environments. Thus, it should be of interest to describe the strength of the C–H⋯O hydrogen bond for constant structural factors so that this intrinsic strength can be quantified and compared to other types of interactions. This description is provided here for *C*_2*h*_-symmetric dimers of acrylic acid by means of the calculations that employ the coupled-cluster theory with singles, doubles, and perturbative triples [CCSD(T)] together with an extrapolation to the complete basis set (CBS) limit. Dimers featuring the C–H⋯O and O–H⋯O hydrogens bonds are carefully investigated in a wide range of intermolecular separations by the CCSD(T)/CBS approach, and also by the symmetry-adapted perturbation theory (SAPT) method, which is based on the density-functional theory (DFT) treatment of monomers. While the nature of these two types of hydrogen bonding is very similar according to the SAPT-DFT/CBS calculations and on the basis of a comparison of the intermolecular potential curves, the intrinsic strength of the C–H⋯O interaction is found to be about a quarter of its O–H⋯O counterpart that is less than one might anticipate.

## 1. Introduction

Hydrogen bonds (HBs) [1] form a particularly important class of noncovalent interactions [2]. From among the numerous types of HBs and related hydrogen-bonding (H-bonding) scenarios [3] (see reference [4] for the most recent survey of important experimental and theoretical research into H-bonding), it is the nature of the C–H⋯O intermolecular H-bonding that is perhaps the most fervently debated (see the important study [5] and work cited therein). This type of H-bonding is known to be quite important. Recent examples of its significance include studies of crystal packing (see the related debate in reference [6]), protein folding [7], structure of certain liquids [8], zeolites [9] and interfaces [10], formation of protein–ligand complexes [11], crosslinking of biomaterials [12], and even the molecular recognition of nerve agents [13]. Hence, in addition to numerous experimental investigations, the C–H⋯O intermolecular interactions were examined by a variety of theoretical approaches. The classic work of Scheiner and his coworkers [14] on the categorization of H-bonding should be mentioned, where the respective properties of the C–H⋯O and “conventional” HBs were compared and the C–H⋯O interactions were found to be “true” HBs (see also the subsequent investigation into the cooperativity of C–H⋯O and O–H⋯O H-bonding [15]). Some of these properties, which are not detailed here, were subsequently considered by a number of computational studies, with the work of Schaefer et al. [16] and Head-Gordon et al. [17] being the most notable examples. Importantly, in a classic paper describing a combination of theory and solid-state NMR experiments [18], it was shown that a distinction should be made between C–H⋯O “contacts” (cases of a small distance between some C–H group and an oxygen atom in crystalline systems) and actual H-bonding intermolecular interactions. Later, an impressive demonstration of this distinction was provided by the combined computational and neutron diffraction (ND) study of polymorphs of glycine [19]. In short, C–H⋯O and N–H⋯O contacts were found in some destabilizing orientations of glycine molecules (details are given in Section 2.1 of this paper). It was thus shown that the residue-specific differences could hide structural motifs that in fact lead to the thermodynamic stability of solid phases [19]. Based on the above considerations, models are devised here with the aim to capture the intrinsic strength of H-bonding interactions. Namely, all structural factors are kept constant except the H-bonding distances in two types of highly symmetric dimers of acrylic acid (AA; systematic name is 2-propenoic acid), as outlined in Section 2.2. Since one type of AA dimers features C–H⋯O, and the other type O–H⋯O HBs, the intrinsic strength of those H-bonding arrangements is quantified by means of high-level quantum chemical calculations. Specifically, in Section 2.3, computations that employ the coupled-cluster theory with singles, doubles, and perturbative triples [CCSD(T)] together with an extrapolation of the energies to the complete basis set (CBS) limit (see the review [20]) are used to follow the dissociation curves. These curves are accurately and consistently fitted to the same type of a modified Dunham expansion (see Equation (3) in Section 4) for the intermonomer separations ranging from 297 to 772 pm, and from 232 to 482 pm, in AA dimers that model C–H⋯O and O–H⋯O H-bonding, respectively. Moreover, in Section 2.4, the symmetry-adapted perturbation theory (SAPT) of intermolecular interactions [21] is combined with the density-functional theory (DFT) treatment of monomers [22] and CBS extrapolations in order to accurately describe the physical nature of noncovalent bonding in the investigated AA dimers. The aforementioned results are discussed in Section 3 and reveal the C–H⋯O interaction to be about four times weaker than its O–H⋯O counterpart in essentially the same structural context. An analogous computational procedure can be used to systematically study other weak interactions and binding motifs that are involved in molecular recognition processes [23].

## 2. Results

### 2.1. Interaction Energies

First, a computational scheme needs to be established for fully reliable predictions of the intermolecular interaction energy, ΔE. The focal-point approach to the CCSD(T)/CBS ΔE estimation was most recently tested [24]. When applied to the interaction energies of systems from the S22 dataset [25], this approach was shown to provide the ΔE values that differed only negligibly from their counterparts published by Sherrill et al. as the S22B collection [26]. Consequently, the same methodology as in reference [24] is used here. Its additional checks were performed for two important systems that feature C–H⋯O intermolecular bonding, namely, benzofuran:formaldehyde [27] and formaldehyde dimers [28]. In particular, the CCSD(T)/CBS ΔE data were obtained for two geometries of the global minimum of the potential-energy surface (PES) of benzofuran:formaldehyde complex, which is a highly challenging system [26]. One geometry was taken from reference [27] (where it is denoted as “semi-experimental” in Supporting Information), while the other, which is pictured in Figure S1, was obtained at the MP2/aTZ level (the second-order Møller–Plesset theory and the standard augmented correlation-consistent polarized valence triple-ζ basis set [29,30]; see Section 4 for further details of the computational methodology adopted in this work). It should be mentioned that rotational constants {A_0_, B_0_, C_0_} were measured in reference [27]. They are {1181, 1096, 788.3} MHz when rounded to four significant digits, and an inspection of Table 1 reveals that the “semi-experimental” structure reproduced these data better than the geometry provided by the MP2/aTZ optimization. However, structural differences led to only a small difference of less than one half of kJ/mol in ΔE values predicted for the two structures (see Table 1). Data in Table 1 also show that the interaction energy estimated in reference [27] by applying the cost-effective “jun-ChS” scheme [31] agrees well with its counterpart obtained in this work. Two additional methods were employed for benchmarking purposes, because these methods were also applied to much larger clusters that feature C–H⋯O and O–H⋯O intermolecular interactions (see Section 3). One of these methods is the DFT-based B2PLYP-D3/def2-QZVPPD approach (the double-hybrid B2-PLYP functional [32,33] combined with the D3 empirical dispersion correction [34] and applied together with the QZVPPD basis set [35]), which is expected to provide highly accurate interaction energies [36]. The other method is the DLPNO-CCSD(T)/CBS technique (the domain-based local pair natural orbital approximation to the CCSD(T) method with an extrapolation to the CBS limit; see Section 4 for pertinent references and computational details). In the following, the B2PLYP-D3/def2-QZVPPD and DLPNO-CCSD(T)/CBS interaction energies are denoted as $\Delta E\left(\mathrm{DFT}\right)$ and $\Delta E\left(\mathrm{DLPNO}\right)$, respectively. An inspection of Table 1 reveals that the interaction energies predicted by various methods for benzofuran:formaldehyde agree with each other very well.

**Figure 1:**
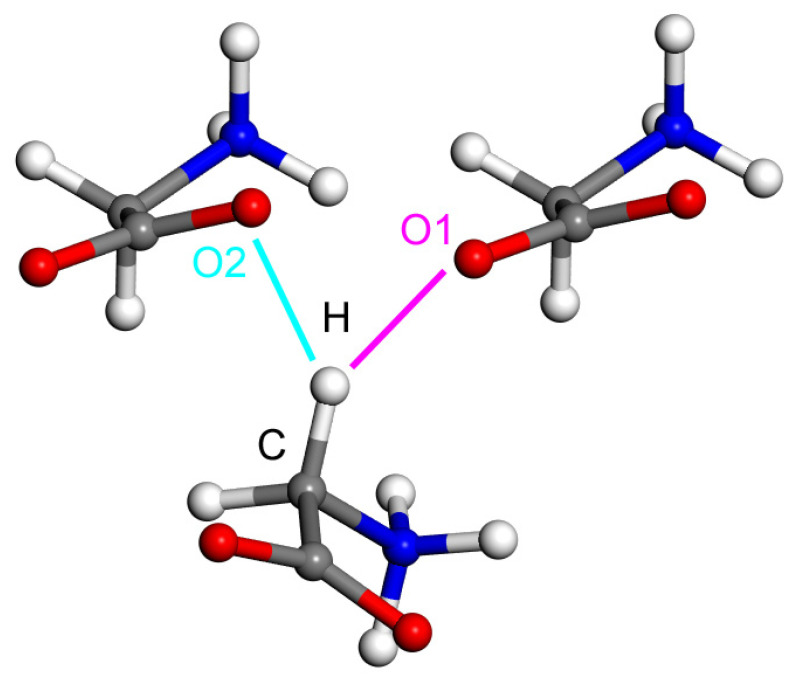
The fragment of ε-glycine in its solid-phase structure [19] that is discussed in the text.

The MP2/aTZ method was further applied to two dimers of formaldehyde, namely, the adducts featuring *C_s_* and *C*_2*h*_ symmetry. The harmonic vibrational zero-point energies, $\Delta {\left(\mathrm{ZPE}\right)}_{h}$, were computed and added to the corresponding ΔE values to estimate the related dissociation energies, ${\left(D_{0}\right)}_{h}$, from ${\left(D_{0}\right)}_{h}=\Delta E$ + $\Delta {\left(\mathrm{ZPE}\right)}_{h}$. The ${\left(D_{0}\right)}_{h}$ data were compared to their counterparts from reference [36] (see Table 2). Expectedly, a close agreement was found, because a very similar computational methodology was adopted in reference [37]. Moreover, the anharmonic effects on zero-point energies were approximated by the VPT2 (vibrational second order perturbation) method at the MP2/aTZ level to obtain the $\Delta {\left(\mathrm{ZPE}\right)}_{a}$ values and combine them with relevant ΔE to assess the anharmonicity-corrected dissociation energies, ${\left(D_{0}\right)}_{a}$, as ${\left(D_{0}\right)}_{a}=\Delta E$ + $\Delta {\left(\mathrm{ZPE}\right)}_{a}$. Results are summarized in Table 2 for the sake of comparison with analogous data presented in reference [38]. Importantly, the two ${\left(D_{0}\right)}_{a}$ estimates for the *C_s_*-symmetric structure, which is the global minimum of the PES of formamide dimer [28], agree within “spectroscopical accuracy” of one kJ/mol (see Table 2). It is noted that the $\Delta E\left(\mathrm{DFT}\right)$ and $\Delta E\left(\mathrm{DLPNO}\right)$ values for this structure are −19.3 and −18.7 kJ, respectively, while for the *C*_2*h*_-symmetric minimum they amount to −15.4 and −15.8 kJ, respectively, and thus are all highly accurate. The dimers of ε-glycine (see Figure 1) are considered in the next paragraph.

The presented procedure for a fully reliable estimation of the canonical CCSD(T)/CBS ΔE was applied to two dimers clipped out from the ND structure of the ε polymorph of glycine [19], which are pictured in Figure 1. The distances between atoms marked H and O2, and H and O1, amount to 218 and 220 pm, respectively. Based on these distances, the C–H⋯O2 contact in the dimer shown in the left in Figure 1 would appear to be even stronger than a similar one in the other dimer, namely, the C–H⋯O1 contact that is present in the dimer appearing in the right side of Figure 1. However, as already shown in reference [19], intermolecular interactions are destabilizing in the arrangement that includes the C–H⋯O2 contact and are stabilizing in the dimer with the C–H⋯O1 contact, which has a longer distance between pertinent proton and oxygen atoms. The ΔE values obtained for these two complexes are +51.8 and −41.1 kJ/mol, respectively, while it is noted that the PIXEL method [39] provided highly accurate values amounting to +51.2 and −40.4 kJ/mol, respectively (see Table 2 of reference [19], where also other geometrical parameters of the C–H⋯O motifs are listed). It is also noted that the $\Delta E\left(\mathrm{DFT}\right)$ and $\Delta E\left(\mathrm{DLPNO}\right)$ results are +52.6 and +53.7 kJ/mol, respectively, for the repulsive arrangement (see references [40,41] for a discussion of repulsive intermolecular interactions), and they amount to −39.2 and −41.1 kJ/mol, respectively, in the stable dimer. It thus can be seen that all computational methods provided very similar interaction energies. Overall, these calculations suggest that the same structural environment needs to be included for a systematic comparison of the strength of various types of HBs. In the following part of the paper, such comparison is presented for the intermolecular C–H⋯O and O–H⋯O H-bonding in a simple model, namely, the AA dimers. At this point, it should be mentioned that the Supplementary Materials files “dimers.xlsx” and “DLPNO.xlsx” contain underlying data for the ΔE and $\Delta E\left(\mathrm{DLPNO}\right)$, respectively, estimation of complexes described so far (benzofuran:formaldehyde and dimers of formamide and ε-glycine).

### 2.2. Model Geometries

**Figure 2:**
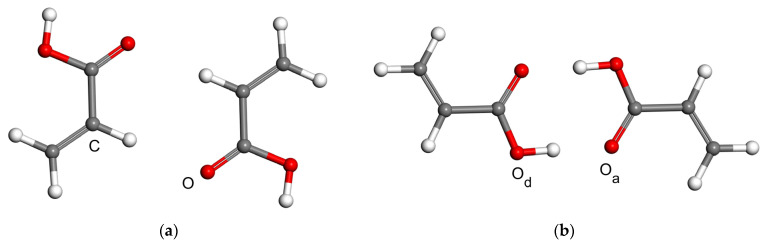
Two types of acrylic acid dimers considered in this work: (**a**) the model for a quantification of the C–H⋯O intermolecular bonding (the distance between carbon atom C and oxygen atom O is followed in calculations discussed in the text); (**b**) the model for a quantification of the O–H⋯O intermolecular bonding (the distance between oxygen atoms marked as O_d_ and O_a_ is followed in calculations discussed in the text).

The acrylic acid dimer (AAD) is one of systems for which the double proton transfer was characterized experimentally (see the review [42]). In particular, the group of Caminati applied microwave spectroscopy measurements to the polar form of AAD and estimated a barrier for the exchange of its hydroxyl protons [43]. Here, nonpolar forms of AAD are considered. Namely, the model for an investigation of C–H⋯O H-bonding has both AA units with *cis* configuration of hydroxyl group relative to vinyl group (see Figure 2a), while that orientation is *trans* in the model describing O–H⋯O interactions (Figure 2b). The full geometry optimization for these two models was carried out at the MP2/aTZ level as in the previous work on molecular complexes [36]. The distance, R, between atoms marked in Figure 2a with “C” and “O”, and in Figure 2b with “O_d_” and “O_a_”, is 341 and 264 pm, respectively, in the pertinent MP2/aTZ minima. All other geometric parameters of these minima can be inferred from their coordinates provided in “min” sheets inside the Supplementary Materials files “CHO.xlsx” and “OHO.xlsx”. It should be noted that in the single-crystal X-ray diffraction structure of AA that features layers of dimers with O–H⋯O hydrogen bonding, the corresponding value of R is 265 pm [44]. The two MP2/aTZ equilibrium geometries were used to vary the parameter R while fixing all other degrees of freedom in order to follow the PES curves, which are described in the next paragraph.

### 2.3. The Interaction Energy Curves

**Figure 3:**
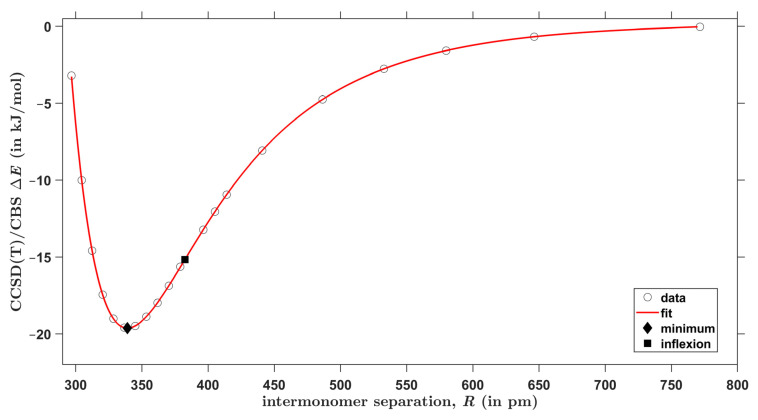
Plot of the distance dependence of the intermolecular interaction energy in acrylic acid dimers that feature C–H⋯O contacts.

**Figure 4:**
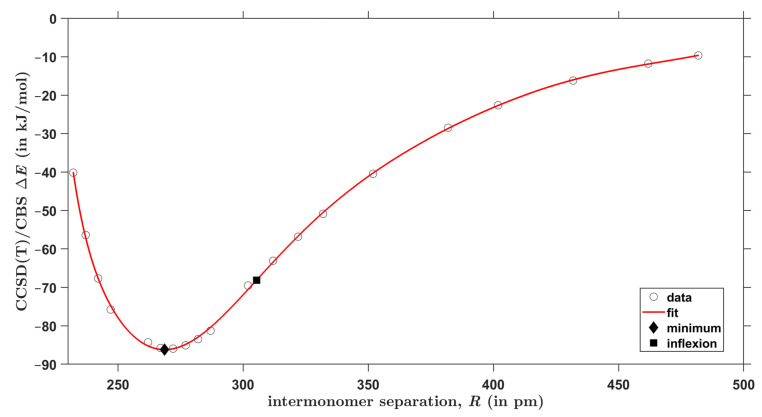
Plot of the distance dependence of the intermolecular interaction energy in acrylic acid dimers that feature O–H⋯O contacts.

For each type of AAD models introduced in Section 2.2, the CCSD(T)/CBS interaction energies were obtained at 20 points in a wide range of the respective intermonomer separation as expressed by the parameter R. There are two equivalent hydrogen bonds in every investigated geometry due to the *C*_2*h*_ symmetry of both types of AA dimers. Figure 3 and Figure 4 graphically present the results for C–H⋯O and O–H⋯O interactions, respectively (all R and ΔE values are collected in the Supplementary Materials Table S1, and the actual geometries and raw energies in “CHO.xlsx” and “OHO.xlsx” spreadsheets). The two sets of ΔE values were accurately fitted to the same functional form, which is specified in Section 4 (further details are given in Table S2). Then, minima were found at distances denoted as $R_{\min.}$. Values of $R_{\min.}$ amount to 339 and 269 pm for C–H⋯O and O–H⋯O models, respectively, and the interaction energy at these points is −19.6 and −86.2 kJ/mol, respectively. These $R_{\min.}$ values are thus in a good agreement with the aforementioned results of the unconstrained MP2/aTZ optimization ($R_{\min.}$ = 341 and 264 pm accordingly). Moreover, the interaction energies at the minima of relaxed geometries are accordingly −19.9 and −85.6 kJ/mol and thus are quite close to their counterparts obtained from the fits of ΔE data. It is worth noting that the fits also enable to estimate a maximal distance at which the investigated contacts become repulsive, that is, a value of R for which ΔE = 0 kJ/mol. This distance is 294 and 226 pm for C–H⋯O and O–H⋯O interactions, respectively. Inflexion points of the fitted curves were located, too, and the corresponding distance between monomers is denoted as $R_{\mathrm{infl}.}$. Namely, $R_{\mathrm{infl}.}$ of 382 and 305 pm, respectively, was found for the curves shown in Figure 3 and Figure 4 (related ΔE amounts to −15.2 and −68.1 kJ/mol, respectively). Positions of these inflexion points are analyzed in terms of the scaled intermolecular distance $R/R_{\min.}$ in Section 3 together with other parameters of the investigated PES. It is worth mentioning that results of the $\Delta E\left(\mathrm{DFT}\right)$ and $\Delta E\left(\mathrm{DLPNO}\right)$ calculations are −20.0 and −19.5 kJ/mol, respectively, for the structure fully optimized by the MP2/aTZ method and featuring C–H⋯O interactions. These values are −87.5 and −85.8 kJ/mol, respectively, for the MP2/aTZ optimized geometry with O–H⋯O H-bonding. It should also be mentioned that the accurately fitted dependencies of ΔE upon R might be of interest in a development and/or testing of force fields [45].

### 2.4. SAPT-DFT Partitioning of the Interaction Energy

**Figure 5:**
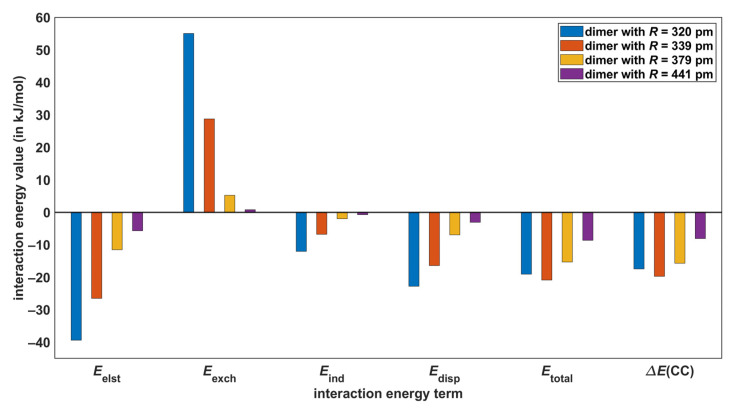
Components of the intermolecular interaction energy extrapolated to the complete basis set limit in models of C–H⋯O hydrogen bonding.

**Figure 6:**
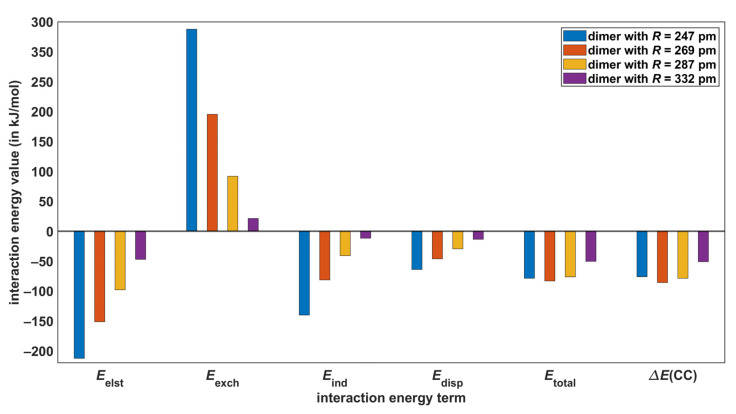
Components of the intermolecular interaction energy extrapolated to the complete basis set limit in models of O–H⋯O hydrogen bonding.

The SAPT-DFT method is a powerful tool for theoretical investigations of noncovalent bonding [46], while it is noted that also the energy decomposition analysis (EDA; see reference [47]) in the DFT framework can be usefully applied to noncovalent interactions [48,49]. The SAPT-DFT/CBS approach, which is specified in Section 4, enables accurate predictions of the intermolecular interaction energy (denoted as $E_{\mathrm{total}}$ in the following). In particular, the root-mean-square deviation between the $E_{\mathrm{total}}$ and corresponding CCSD(T)/CBS data (denoted as $\Delta E\left(\mathrm{CC}\right)$ in the following) for a diverse set of 18 dimers was found to be as low as 0.84 kJ/mol [24]. At the same time, the SAPT-DFT technique partitions a $E_{\mathrm{total}}$ value into well-defined contributions that stem from electrostatic, exchange, induction, and dispersion interactions [50]. These components of the total interaction energy are denoted here as $E_{\mathrm{elst}}$, $E_{\mathrm{exch}}$, $E_{\mathrm{ind}}$, and $E_{\mathrm{disp}}$, respectively, with $E_{\mathrm{total}}$ = $E_{\mathrm{elst}}+E_{\mathrm{exch}}+E_{\mathrm{ind}}+E_{\mathrm{disp}}$ (see Section 4). Their dependence upon the distance R in AA dimers was followed (all underlying values are collected in Table S3). Results are presented in Figure 5 and Figure 6, where also pertinent $\Delta E\left(\mathrm{CC}\right)$ data are included for comparison purposes. The key values are listed in Table 3 and reveal that the biggest absolute difference between the reference $\Delta E\left(\mathrm{CC}\right)$ results and their $E_{\mathrm{total}}$ counterparts is 2.8 kJ/mol and occurs in the model of O–H⋯O interactions with R = 247 pm (the relative difference of these interaction energies is 3.7%), while the highest relative difference amounts to 9.2% (in the case of the C–H⋯O model with R = 320 pm). The present SAPT-DFT/CBS data can thus be considered to be reliable throughout the investigated ranges of intermonomer separations and are analyzed below.

Figure 5 and Figure 6 show that the $E_{\mathrm{elst}}$ energy, which arises in the first order of the perturbation theory of intermolecular interactions, is the highest stabilizing (that is, the most negative) contribution to all $E_{\mathrm{total}}$ values. The $E_{\mathrm{exch}}$ term is also the first-order contribution and surpasses $E_{\mathrm{elst}}$ at the two shorter distances in models of both C–H⋯O and O–H⋯O interactions. Consequently, higher-order terms are responsible for binding in these four complexes [51]. At the two longer distances, however, the first-order terms alone would lead to an attractive interaction, as the sums $\left(E_{\mathrm{elst}}+E_{\mathrm{exch}}\right)$ are negative (see Figure 5 and Figure 6, and also Table S3). In the computational approach adopted here, $E_{\mathrm{disp}}$ component combines the second-order dispersion contributions, and $E_{\mathrm{ind}}$ adds the second-order induction contributions and an estimate of all higher-order terms [52]. The $E_{\mathrm{ind}}$ energy is a bit more important than $E_{\mathrm{disp}}$ in AA dimers that represent O–H⋯O H-bonding, and conversely $E_{\mathrm{disp}}$ contributes slightly more than $E_{\mathrm{ind}}$ to a stabilization of the C–H⋯O models. Nevertheless, adding $E_{\mathrm{ind}}$ energies to $\left(E_{\mathrm{elst}}+E_{\mathrm{exch}}\right)$ sums would result in a stabilization of all investigated dimers. This would not hold for $E_{\mathrm{disp}}$ contribution, as it amounts to −64.0 kJ/mol in the O–H⋯O model with R = 247 pm and does not offset $\left(E_{\mathrm{elst}}+E_{\mathrm{exch}}\right)$ of 125.4 kJ/mol, contrary to $E_{\mathrm{ind}}$ of −140.0 kJ/mol obtained in this case (see Table S3). The dispersion-to-electrostatics ratio provided by SAPT calculations is a parameter that is frequently employed for the categorization of noncovalently bound complexes [53]. These ratios obtained from the SAPT-DFT/CBS approach are listed in Table 3. They all fall into the range of values that are typical for HBs [54]. Overall, the electrostatics appear to be the most important contribution to the intermolecular bonding in both types of AA dimers considered here.

For the intermonomer separations considered in the previous paragraph, also the EDA calculations were performed. The implementation in the Amsterdam Modeling Suite [55] of the EDA approach from reference [56] was applied together with the B3LYP [57,58,59] combination of DFT functionals, the aforementioned D3 empirical dispersion correction, and the QZ4P basis set [60] (further details are given in Section 4). Resulting B3LYP-D3/QZ4P data are collected in Table S4 for both investigated H-bonding types. These data demonstrate the expected distance dependences of the interaction energy terms, which are graphically presented in Figure S2.

## 3. Discussion

**Figure 7:**
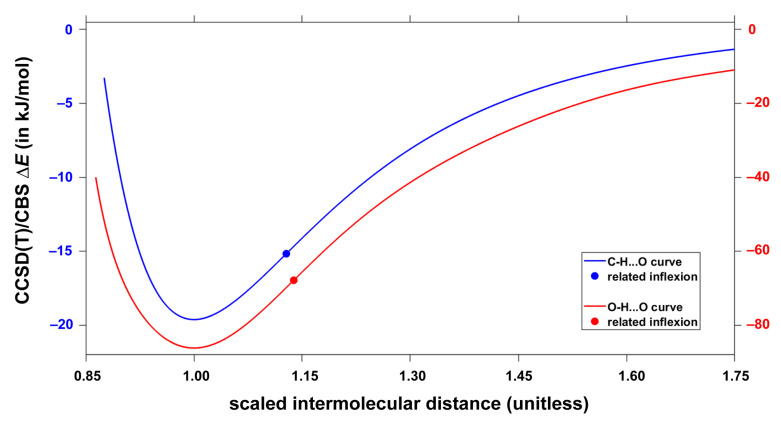
Dependence of the intermolecular interaction energy on the relative distance between monomers in models of C–H⋯O (*y*-axis to the left; upper curve) and O–H⋯O (*y*-axis to the right; lower curve) hydrogen bonding.

The most recent review article [61] stressed the importance of a proper denomination of weak interactions in various kinds of aggregates. For a quantification of multitude of intermolecular contacts, it should be beneficial to apply high-level quantum chemical calculations to models that avoid competing structural effects [62]. This way the intrinsic strength of the respective types of noncovalent bonding can be obtained. Here, the CCSD(T)/CBS approach was used to predict the intermolecular interaction energy as a function of the C–H⋯O or O–H⋯O H-bonding distances in analogous environments provided by the topology of acrylic acid dimers (see Section 2.2 and Section 2.3). Accurate fits of resulting ΔE to the same functional form were obtained and enable a side-by-side comparison of the two interactions. It is convenient for such comparison to employ the relative distance, r, $r=R/R_{\min.}$ with the respective $R_{\min.}$ values specified in Section 2.3. Figure 7 depicts the dependence of two sets of interaction energies upon the same r data ranging from about 0.86 up to 1.75. In relative terms, the two curves are very similar (see Figure 7). In particular, inflexion points of the C–H⋯O and O–H⋯O models lie at r = 1.128 and 1.138, respectively. The two ΔE sets, however, span completely different intervals of absolute values in the investigate range of r. For instance, ΔE = −15.0 kJ/mol for the C–H⋯O model is reached at around r = 0.924 in the descending part of the dissociation curve, while at this relative distance the O–H⋯O model features ΔE as high as −76.1 kJ/mol. Additionally, at $R_{\min.}\;$ and $R_{\mathrm{infl}.}$ distances, the interaction energy of the O–H⋯O arrangement is over four times higher than exhibited by its C–H⋯O counterpart (see Section 2.3 for details). Consequently, the intrinsic strength of the C–H⋯O interaction is much lower than could be anticipated on the basis of, for example, experimental estimates of interaction energies in fragments of crystalline austdiol (C_12_H_12_O_5_) featuring either C–H⋯O or O–H⋯O HBs [63], and the ΔE data obtained for *N*-methylacetamide:dimethylformamide dimers containing either C–H⋯O or classical N–H⋯O HBs [64]. This finding is in line with conclusions of the most recent computational study of host–guest interactions, where the C–H⋯O H-bonding was found to be weak, yet very important for the thermodynamic stabilization of certain complexes [65].

**Figure 8:**
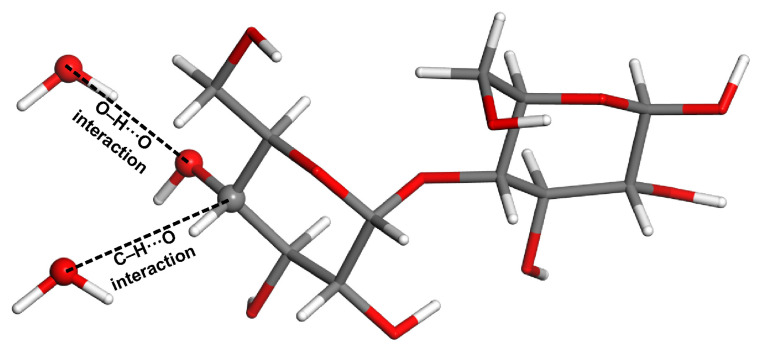
The model of C–H⋯O and O–H⋯O interactions in *β*-maltose investigated by calculations.

It should be of interest to reproduce the aforementioned finding in some more complex systems. Consequently, using the coordinates of *β*-maltose from the ND study [66], the cluster was prepared in order to study C–H⋯O and O–H⋯O H-bonding separately, but in the same structural environment (see Figure 8). Two dimers are thus considered that both contain one *β*-maltose and one water molecule. Respective water molecules replace the neighboring *β*-maltose featuring either C–H⋯O interaction (in this case the C–O distance is 366 ppm) or O–H⋯O interaction (the distance between pertinent oxygen atoms is 277 pm). Due to the size of these dimers (48 atoms), the canonical CCSD(T)/CBS calculations of the interaction energy would be impractical. Hence, the $\Delta E\left(\mathrm{DLPNO}\right)$ values were obtained instead. They amount to −4.9 and −17.2 kJ/mol for the model of C–H⋯O and O–H⋯O interaction, respectively (the corresponding $\Delta E\left(\mathrm{DFT}\right)$ results are −4.4 and −16.8 kJ/mol). These data show that the intrinsic strength of the C–H⋯O interaction is about a quarter of its O–H⋯O counterpart.

**Figure 9:**
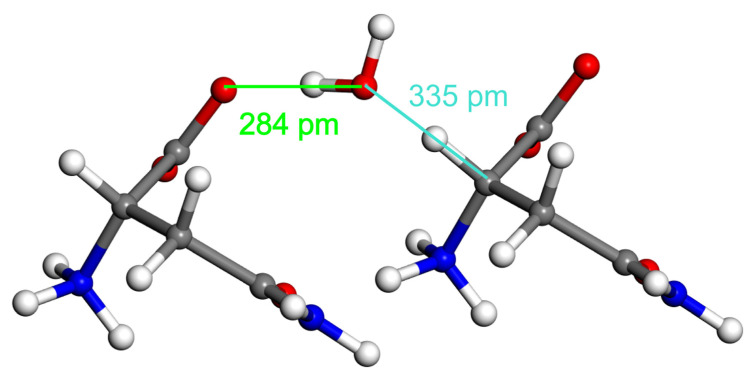
The fragment of crystalline L-asparagine monohydrate employed for a quantification of two types of hydrogen bonding, namely, O–H⋯O (shown in green) and C–H⋯O (turquoise).

Furthermore, the present theoretical approach was used in order to compare C–H⋯O and O–H⋯O H-bonding arrangements that involve a crystalline water molecule. The ND structure of L-asparagine monohydrate [67] is suitable for such a comparison. As shown in Figure 9, both C–H⋯O and O–H⋯O HBs are formed by the water that bridges L-asparagine molecules, which are positioned approximately in a direction of the crystallographic *a* axis of the structure from reference [67]. Hence, clusters that contain this water molecule and the pertinent L-asparagine were prepared and the ΔE, $\Delta E\left(\mathrm{DLPNO}\right)$, and $\Delta E\left(\mathrm{DFT}\right)$ values were established while employing the ND geometry. Results are summarized in Table 4 (the underlying absolute energies are available from related Excel spreadsheets included in the Supplementary Materials). As expected, the interaction energy values computed by the three high-level methods agree with each for both types of H-bonding. Data in Table 4 reconfirm the finding presented above, as they show the O–H⋯O interaction to be about four times stronger than its C–H⋯O counterpart in essentially the same structural environment.

## 4. Materials and Methods

All the MP2/aTZ geometry optimizations and estimations of vibrational zero-point energies were performed using the Gaussian 16, revision C.01 suite of codes [68] with default settings. This version of the Gaussian program package was also used to obtain the B2PLYP-D3(BJ)/def2-QZVPD interaction energies.

The CCSD(T)/CBS interaction energies were obtained by the focal-point method expressed by Equation (1) (see reference [24] for further details):

(1) $$\Delta E_{\mathrm{CCSD}\left(T\right)}^{\mathrm{CBS}}=\Delta E_{\mathrm{HF}}^{a5Z}+\Delta E_{\mathrm{MP}2}^{a5Z}+\Delta E_{\mathrm{post}-\mathrm{MP}2}^{\mathrm{aTZ}}$$

where subscripts denote the respective energy terms, namely, the total Hartree–Fock energy (HF), the MP2 correlation energy (MP2), and the higher-order correlation energy (post-MP2), and superscripts specify the basis set used to compute the respective term. The MP2/a5Z correlation energies were obtained in the resolution-of-the-identity integral approximation [69,70] while using the relevant auxiliary basis sets [70]. Calculations of the HF/a5Z and MP2/a5Z energies were performed in Turbomole, version 7.1 [71]. Calculations of the canonical CCSD(T)/aTZ and MP2/aTZ correlation energies were carried out in Molpro 2021.2 [72].

Additionally, in Molpro 2021.2, the SAPT-DFT/CBS interaction energies were estimated using the same procedures as in our most recent work [36]. The $E_{\mathrm{elst}}$, $E_{\mathrm{exch}}$, $E_{\mathrm{disp}}$, and $E_{\mathrm{ind}}$ contributions to the total interaction energy, $E_{\mathrm{total}}$, from Section 2.4 are related to the underlying interaction energy terms as follows: $E_{\mathrm{elst}}\;$ and $E_{\mathrm{exch}}$ are the polarization and exchange energy contributions, respectively, arising in the first order of the perturbation theory of intermolecular interactions [73]; $E_{\mathrm{disp}}$ is the dispersion energy contribution obtained as a sum of the second order terms $E_{\mathrm{disp}.}^{\mathrm{SAPT}\;\left(2\right)}$ and $E_{\mathrm{disp}.-\mathrm{exch}.}^{\mathrm{SAPT}\;\left(2\right)}$ [74]; and $E_{\mathrm{ind}}$ is the induction energy contribution approximated by a sum of the second order terms $E_{\mathrm{ind}.-\mathrm{exch}.}^{\mathrm{SAPT}\;\left(2\right)}$ and $E_{\mathrm{ind}.}^{\mathrm{SAPT}\;\left(2\right)}$ [75] and the correction term $E_{\delta \left(\mathrm{HF}\right)}^{\mathrm{SAPT}\;}$, which is computed at the HF level [51].

DLPNO-CCSD(T)/CBS interaction energies were estimated by the focal-point method from reference [24], which applies Equation (2) (the notation is as in Equation (1), and right arrow is used to indicate an application of the two-point extrapolation formula from reference [76]):

(2) $$\Delta E_{\mathrm{DLPNO}-\mathrm{CCSD}\left(T\right)}^{\mathrm{CBS}}=\Delta E_{\mathrm{HF}}^{\mathrm{aQZ}}+\Delta E_{\mathrm{MP}2}^{\mathrm{aTZ}\to \mathrm{aQZ}}+\Delta E_{\mathrm{post}-\mathrm{MP}2}^{\mathrm{aTZ}\to \mathrm{aQZ}}$$

while the CCSD(T) and MP2 correlation energies were obtained in the DLPNO approximation [77,78,79,80]. The ORCA 5.0.3 program package [81] was used with the “TightPNO” set of parameters for the truncation of the electron-correlation space and with the default method of the orbital localization.

The least-squares fits of $\Delta E\left(R\right)$ data employed the following functional form:

(3) $$\Delta E\left(R;\;r_{e},a_{0},a_{1},a_{2},a_{3},a_{4},a_{5},a_{6},V_{e}\right)=a_{0}\xi^{2}\left(1+a_{1}\xi +a_{2}\xi^{2}+a_{3}\xi^{3}+a_{4}\xi^{4}+a_{5}\xi^{5}+a_{6}\xi^{6}\right)+V_{e}$$

with $\xi =\left(R-r_{e}\right)/R$ and R as defined in Section 2.2. The Levenberg–Marquardt algorithm from the “lsqcurvefit” function of MATLAB^®^ Optimization Toolbox™ was applied.

The B3LYP-D3/QZ4P EDA calculations were performed in the AMS version 2022.103 [55]. In these calculations, core orbitals were not frozen, and the “Good” convergence thresholds were applied.

## 5. Conclusions

More than 20 years ago, it was found that in a certain context the C–H⋯O H-bonding could be even stronger than the O–H⋯O interaction [82]. However, a computational procedure proposed here, which is based on an analysis of the CCSD(T)/CBS data obtained for accurate geometries, shows that C–H⋯O HBs are intrinsically weaker than O–H⋯O HBs by a factor of about four. This procedure should be applied to systematically study other noncovalent interactions to assess their role in various types of intermolecular association processes [83,84].

## Figures and Tables

**Table 1:** Geometrical parameters and interaction energies of the global minimum of benzofuran:formaldehyde complex.

Structure	Rotational Constants/MHz			ΔE/kJ/mol	$\Delta E\left(\mathrm{DFT}\right)/\mathrm{kJ}/\mathrm{mol}$	$\Delta E\left(\mathrm{DLPNO}\right)/\mathrm{kJ}/\mathrm{mol}$
	A	B	C			
optimized by MP2/aTZ	1235	1174	841.7	−15.28	−14.64	−14.73
semi-experimental ^1^	1180	1103	789.0	−15.58 (−16.15) ^2^	−15.36	−15.08

^1^ See reference [27] for details. ^2^ Obtained in reference [31] using “jun-ChS” scheme.

**Table 2:** Energies of the formamide dimer of the *C_s_* symmetry and (in parentheses) *C*_2*h*_ symmetry. All values are in kJ/mol.

Parameter	Optimized Structure		
	MP2/aTZ (This Work)	MP2/haTZ (Ref. [37])	MP2/aQZ (Ref. [38])
ΔE	−19.1 ^1^ (−16.0) ^1^	−18.7 ^2^ (−15.5) ^2^	−19.2 ^3^
$\Delta {\left(\mathrm{ZPE}\right)}_{h}$	7.1 ^1^ (4.3) ^1^	6.9 ^4^ (4.1) ^4^	—
$\Delta {\left(\mathrm{ZPE}\right)}_{a}$	6.2 ^1^ (3.7) ^1^	—	5.5 ^5^
${\left(D_{0}\right)}_{h}$	−12.0 ^1^ (−11.7) ^1^	−11.8 (−11.4)	—
${\left(D_{0}\right)}_{a}$	−12.9 ^1^ (−12.3) ^1^	—	−13.7

^1^ Obtained as described in the text. ^2^ CCSD(T)-F12/haTZ value from Table S2 of reference [37]. ^3^ CCSD(T)-F12/a5Z value from Table 3 of reference [38]. ^4^ MP2/haTZ value from Table 2 of reference [37]. ^5^ The “semi-empirical” value from reference [38].

**Table 3:** The distance dependence of intermolecular interaction energies.

Interaction	Distance, R/pm	$\Delta E\left(\mathrm{CC}\right)/\mathrm{kJ}/\mathrm{mol}$	$E_{\mathrm{total}}/\mathrm{kJ}/\mathrm{mol}$	$\frac{E_{\mathrm{disp}}}{E_{\mathrm{elst}}}$
C–H⋯O	320	−17.5	−19.1	0.580
	329 ^1^	−19.7	−20.9	0.620
	379	−15.7	−15.3	0.599
	441	−8.1	−8.7	0.543
O–H⋯O	247	−75.8	−78.6	0.302
	269 ^2^	−85.6	−83.1	0.304
	287	−78.7	−76.1	0.303
	332	−50.9	−50.3	0.284

^1^ Minimum of the curve shown in Figure 3. ^2^ Minimum of the curve shown in Figure 4.

**Table 4:** Interaction energies of two types of dimers formed between L-asparagine and water. All values are in kJ/mol.

Hydrogen Bonding Type	ΔE	$\Delta E\left(\mathrm{DLPNO}\right)$	$\Delta E\left(\mathrm{DFT}\right)$
C–H⋯O	−13.12	−12.91	−12.34
O–H⋯O	−49.09	−48.88	−48.79

## Data Availability

The data presented in this study are available in the article and in the Supplementary Materials.

## Supplementary Materials

The following supporting information can be downloaded at: https://www.mdpi.com/article/10.3390/molecules28114478/s1, Table S1: Data plotted in Figure 3 and Figure 4; Table S2: Parameter values as obtained from the fits using Equation (3); Table S3: Raw data used to create Figure 5 and Figure 6; Figure S1: The MP2/aTZ minimum of the benzofuran:formaldehyde adduct; Table S4: Results of the EDA calculations; Figure S2: The distance-dependence of the EDA terms; Excel spreadsheets that are described in Section 2: “dimers.xlsx”, “CHO.xlsx”, “OHO.xlsx”, and “DLPNO.xlsx”.
