# Supplementary material for: Quantifying the Intrinsic Strength of C–H⋯O Intermolecular Interactions
Source: Molecules. 2023 May 31;28(11):4478. doi: 10.3390/molecules28114478 (PMC10254764; doi:10.3390/molecules28114478)

Supplementary Materials to “Quantifying the Intrinsic Strength of C–H···O Intermolecular Interactions” by Czernek, Brus, Czerneková & Kobera (*Molecules* 2023)

Table S1. Data plotted in Figures 3 and 4.

model of the C–H···O interaction (Fig. 3)		model of the O–H···O interaction (Fig. 4)	
R (in pm, rounded)	ΔE (in J/mol, rounded)	R (in pm, rounded)	ΔE (in kJ/mol, rounded)
297	–3209	232	–40169
304	–10012	237	–56390
312	–14593	242	–67697
320	–17456	247	–75783
328	–19013	262	–84302
337	–19592	267	–85781
345	–19484	272	–85949
353	–18887	277	–85097
362	–17981	282	–83472
370	–16872	287	–81281
379	–15635	302	–69539
396	–13230	312	–63090
405	–12054	322	–56808
414	–10948	332	–50879
441	–8078	352	–40456
486	–4756	382	–28497
533	–2767	402	–22617
580	–1584	432	–16213
646	–676	462	–11813
772	–32	482	–9673
339 ($R_{\text{min.}}$)	–19623	269 ($R_{\text{min.}}$)	–86213
382 ($R_{\text{infl.}}$)	–15169	305 ($R_{\text{infl.}}$)	–68116

Table S2. Parameter values as obtained from the fits using Equation 3.

model of the C–H···O interaction (Fig. 3)		model of the O–H···O interaction (Fig. 4)	
parameter	value	parameter	value
r_e	3.39043328292679e+000 pm	r_e	2.68604794773183e+000 pm
a_0	523.886090663663e+000	a_0	1.51172306772681e+003
a_1	−3.42441068460830e+000	a_1	−224.844260019574e−003
a_2	3.46216907307006e+000	a_2	−9.65395289509222e+000
a_3	3.85853134561235e+000	a_3	−35.5936125964515e+000
a_4	−11.8036418433260e+000	a_4	350.263400321026e+000
a_5	8.52097907943845e+000	a_5	−804.060973259444e+000
a_6	−1.13195550727040e+000	a_6	605.364663753520e+000
V_e	−19.6225847619089e+000 kJ/mol	V_e	−86.2134539138866e+000 kJ/mol

Table S3. Raw data used to create Figures 5 and 6. All interaction energy terms are in kJ/mol. Shown in Figures 5 and 6 are:

$E_{\mathrm{elst}} = E^{\mathrm{SAPT\ (1)}}_{\mathrm{elst.}}$; $E_{\mathrm{exch}} = E^{\mathrm{SAPT\ (1)}}_{\mathrm{exch.}}$; $E_{\mathrm{ind}} = E^{\mathrm{SAPT\ (2)}}_{\mathrm{ind.}} + E^{\mathrm{SAPT\ (2)}}_{\mathrm{ind.-exch.}} + E^{\mathrm{SAPT}}_{\delta(\mathrm{HF})}$; $E_{\mathrm{disp}} = E^{\mathrm{SAPT\ (2)}}_{\mathrm{disp.}} + E^{\mathrm{SAPT\ (2)}}_{\mathrm{disp.-exch.}}$; $E_{\mathrm{total}} = E_{\mathrm{elst}} + E_{\mathrm{exch}} + E_{\mathrm{ind}} + E_{\mathrm{disp}}$;

and $\Delta E(\mathrm{CC})$.

interaction energy term	model of the C–H···O interaction; R is specified right below (in pm)				model of the O–H···O interaction; R is specified right below (in pm)			
	320	339 ($R_{\mathrm{min.}}$)	379	441	247	269 ($R_{\mathrm{min.}}$)	287	332
$E^{\mathrm{SAPT\ (1)}}_{\mathrm{elst.}}$	−39.3350	−26.4585	−11.5892	−5.6835	−212.2520	−151.2490	−97.8784	−46.9350
$E^{\mathrm{SAPT\ (1)}}_{\mathrm{exch.}}$	55.0872	28.7655	5.2057	0.8048	337.6787	195.5275	92.0015	21.5863
$E^{\mathrm{SAPT\ (2)}}_{\mathrm{disp.}}$	−20.4710	−10.6282	−2.4709	−0.7624	−214.4968	−122.3079	−58.2948	−15.4007
$E^{\mathrm{SAPT\ (2)}}_{\mathrm{disp.-exch.}}$	14.0993	6.6182	0.9120	0.1162	136.7242	76.2138	33.8666	7.1074
$E^{\mathrm{SAPT\ (2)}}_{\mathrm{ind.}}$	−28.1217	−19.5323	−7.6629	−3.2283	−86.0319	−60.5658	−37.7111	−15.7542
$E^{\mathrm{SAPT\ (2)}}_{\mathrm{ind.-exch.}}$	5.3224	3.1275	0.7230	0.1423	22.0241	14.6017	8.0481	2.4037
$E^{\mathrm{SAPT}}_{\delta(\mathrm{HF})}$	−5.6397	−2.7518	−0.4214	−0.0545	−62.2469	−35.2823	−16.1671	−3.3206
E_{total}	−19.0585	−20.8594	−15.3035	−8.6654	−78.6006	−83.0616	−76.1350	−50.3132
$\Delta E(\mathrm{CC})$	−17.4561	−19.7275	−15.6749	−8.0781	−75.7832	−85.6417	−78.6820	−50.8790

six pages in total

Figure S1. The MP2/aTZ minimum of the benzofuran:formaldehyde adduct.

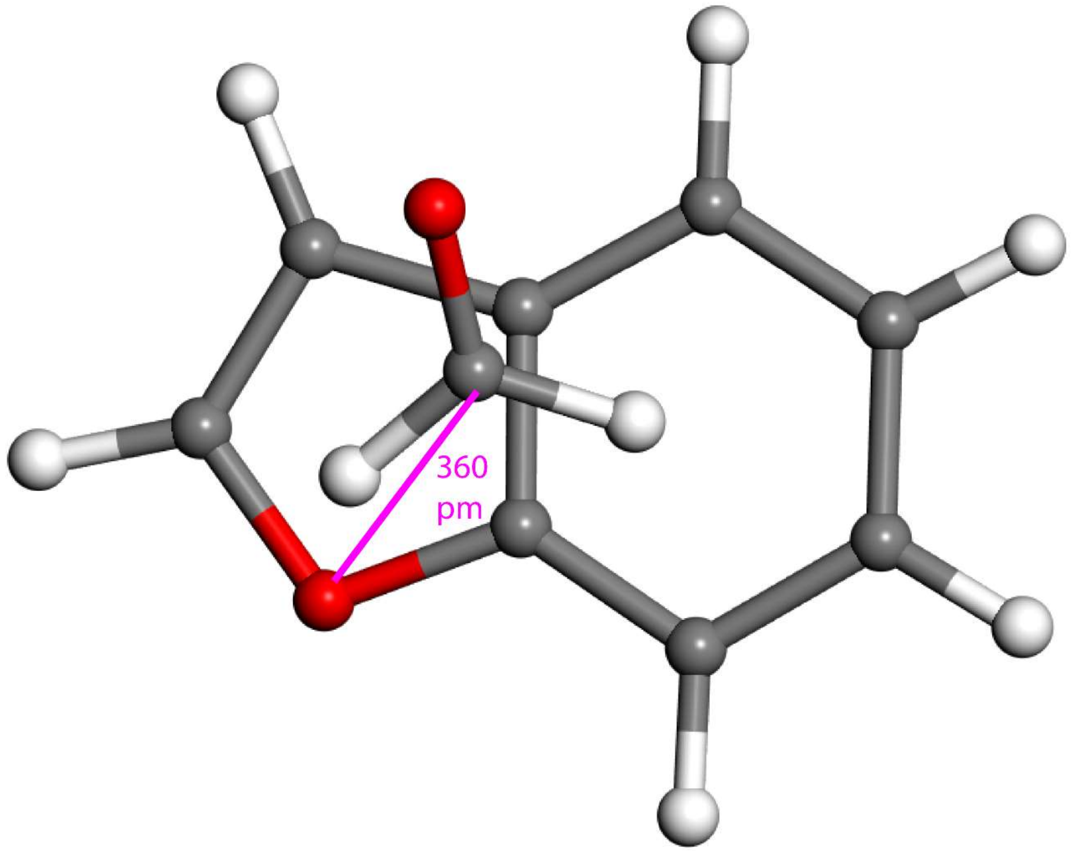

Table S4. Results of the EDA calculations performed at the B3LYP-D3/QZ4P level. Respective terms are: Pauli repulsion, ΔE_{Pauli} (see Eq. 13 of ref. [55]; the electrostatic interaction, ΔE_{elstat} (see Eq. 11 of ref. [55]); the orbital interaction, ΔE_{oi} (see Eq. 22 of ref. [55]); the empirical dispersion correction, ΔE_{D3} (see ref. [34]); $\Delta E(\text{EDA}) = \Delta E_{\text{Pauli}} + \Delta E_{\text{elstat}} + \Delta E_{\text{oi}} + \Delta E_{\text{D3}}$; the basis set superposition error correction, $\Delta E(\text{BSSE})$; and $\Delta E(\text{DFT1}) = \Delta E(\text{EDA}) + \Delta E(\text{BSSE})$. Also shown is $\Delta E(\text{CC})$ as in Table S3.

interaction energy term	model of the C–H···O interaction; R is specified right below (in pm)				model of the O–H···O interaction; R is specified right below (in pm)			
	320	339 ($R_{\text{min.}}$)	379	441	247	269 ($R_{\text{min.}}$)	287	332
ΔE_{Pauli}	46.75	26.49	4.16	1.01	319.07	178.82	95.37	9.03
ΔE_{elstat}	−39.61	−28.46	−11.44	−5.56	−214.77	−152.53	−107.58	−34.95
ΔE_{oi}	−16.37	−10.44	−2.66	−0.87	−174.46	−106.47	−63.80	−10.68
ΔE_{D3}	−10.61	−9.97	−5.41	−2.67	−11.63	−11.04	−10.31	−6.87
$\Delta E(\text{EDA})$	−19.84	−22.38	−15.36	−8.09	−81.80	−91.22	−86.32	−43.47
$\Delta E(\text{BSSE})$	−0.30	−0.24	−0.14	−0.06	−0.84	−0.70	−0.58	−0.30
$\Delta E(\text{DFT1})$	−20.04	−22.62	−15.50	−8.15	−82.64	−91.92	−86.90	−43.77
$\Delta E(\text{CC})$	−17.46	−19.73	−15.67	−8.08	−75.78	−85.64	−78.68	−50.88

Figure S2. The dependence of interaction energies from Table S4 upon the intermonomer separation *R* in acrylic acid dimers that model the C–H···O hydrogen bonding.

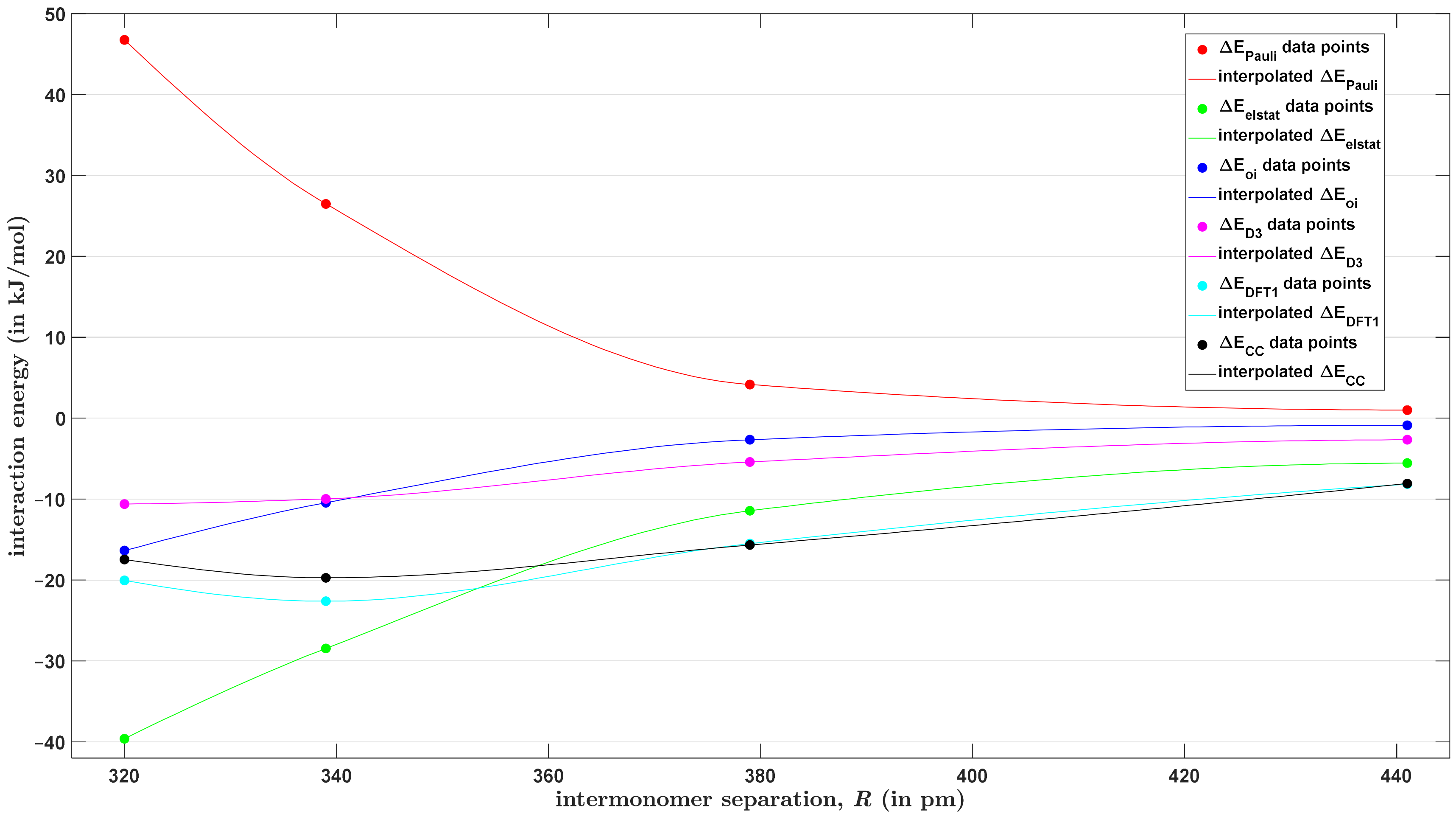

Supplement: Supplementary file 1 [file molecules-28-04478-s001.zip › SI.pdf]
